# Leakage Current Analysis of Glass, Porcelain, and Silicone Insulators Under Icing Conditions Using Spectrogram-Based Deep Convolutional Neural Networks

**DOI:** 10.3390/s26134121

**Published:** 2026-06-30

**Authors:** Muhammed Buğracan Özküçük, Ömer Faruk Alçin, Muhsin Tunay Gençoğlu

**Affiliations:** 1 Department of Electrical and Electronics Engineering, Faculty of Engineering and Natural Sciences, Malatya Turgut Ozal University, 44210 Malatya, Türkiye; bugracan.ozkucuk@ozal.edu.tr; 2 Department of Software Engineering, Faculty of Engineering, Inonu University, 44210 Malatya, Türkiye; omer.alcin@inonu.edu.tr; 3 Department of Electrical and Electronics Engineering, Faculty of Engineering, Firat University, 23119 Elazığ, Türkiye

**Keywords:** iced insulator, leakage current, signal processing, spectrogram, CNN

## Abstract

Insulators are essential for the secure and uninterrupted functioning of high-voltage transmission lines. However, since insulators are exposed to the outdoor environment, they are inevitably affected by environmental conditions such as icing. Accumulation of ice on insulator surfaces adversely impacts insulation efficacy and elevates surface leakage currents, resulting in power outages. This research presents a spectrogram-based convolutional neural network (CNN) model for identifying icing conditions on the surfaces of glass, porcelain, and silicone insulators. Insulators are labeled in three classes under laboratory conditions: ice-free, slightly iced (t < 12 mm), and iced (t > 20 mm). High voltage was applied at three distinct levels ranging from 10 to 50 kV, considering the icing conditions of each insulator, and leakage current signals were recorded. The Butterworth and smoothing filters were first applied to the leakage current signals, which were then transformed into spectrogram images using the Fourier transform and used as input for the created CNN architecture. Additionally, spectrogram images were also applied to AlexNet, GoogLeNet, and ResNet-50 architectures. The suggested CNN architecture attained an accuracy of 97.78% to 100% across all operating situations for glass and silicone insulators while demonstrating a classification success rate of 82.22% to 100% for porcelain insulators. Experiments indicate that the accuracy rates of established models in the literature (AlexNet, GoogLeNet, and ResNet-50) diminished to as low as 73%, particularly in porcelain insulator data, thereby validating the developed model’s proficiency in differentiating during icing detection processes and its adaptability to varying conditions.

## 1. Introduction

The accelerating pace of technological advancement has rendered electrical energy essential to human existence. Therefore, the safe and uninterrupted transmission of electrical energy is of critical importance for the sustainability of modern societies. In this transmission process, insulators have become a fundamental component of power systems by ensuring electrical integrity through insulation between the conductor and the pole while also providing mechanical support.

Insulators predominantly function in exposed environments and under severe conditions, rendering them perpetually influenced by environmental elements. Atmospheric ice is regarded as one of the most significant environmental hazards concerning power systems [1,2]. In the study conducted for ice thickness estimation, parameters such as temperature, humidity, wind speed, wind direction, and precipitation were selected as inputs [3]. A hybrid model combining the snake optimization (ISO) algorithm, deep extreme learning machine (DELM), and hybrid kernel extreme learning machine (HKELM) has been proposed. Although the hybrid method has shown superior performance, it needs to be improved, as the study is carried out under ideal conditions and is dependent on meteorological conditions. The study, categorized into six distinct groups according to ice thickness, employed CNN and SVM methodologies [4]. An accuracy rate of 90% was attained. The gray-level co-occurrence matrix (GLCM) method was used to extract and classify surface feature patterns during flashover events in an ice-covered string insulator [5]. Four feature characteristics were employed to delineate the various stages of the flashover event: angular second moment (ASM), contrast (CON), inverse difference moment (IDM), and entropy. A weakly supervised and progressive transfer learning method has been proposed to recognize different types of insulator and ice, such as snow, rime, mixed rime, icing, and normal [6]. The method’s precision, recall, and mean average precision were determined to be 86.6%, 91.3%, and 90.1%, respectively. A deep learning model based on the Large Dynamic Core Acquisition Network (LDKA-NET) has been proposed for the detection of iced-over transmission lines under different weather conditions [7]. The critical voltage level of insulators falls with increasing icing severity. Power outages could result from this circumstance [8]. Developing high-precision models for predicting ice thickness and flashover threats is essential for managing these risks.

A prevalent method for monitoring ice conditions and making forecasts is the examination of leakage current (LC) data [1,8,9,10]. Using wavelet analysis, the leakage currents of de-noised iced insulators were analyzed [9]. The ice surface discharge mode was shown to be associated with the low-frequency components of leakage currents, whereas the air gap discharge mode is associated with the medium- and high-frequency components.

Convolutional Neural Network (CNN)-based deep learning methods have gained prominence in detecting not only ice formation on insulators but also various insulator defects such as contamination and breakage in recent years [11,12,13]. In the study conducted to detect insulators with different aspect ratios, a method based on Faster R-CNN was proposed [14]. The Region Proposal Network (RPN) and the Non-Maximum Suppression (NMS) algorithm have been developed to provide compatibility across varying scales. The model’s average precision (AP) value was determined to be 0.818. A cross-domain multi-level feature adaptive R-CNN (CMFAA R-CNN) model has been proposed for the detection of insulator defects [15]. Experimental results show that the proposed method improved AP50 performance by 8.6% compared with the Faster R-CNN model. A CNN-LSTM-based system that uses signals from an acoustic sensor and an ultra-high-frequency (UHF) antenna together has been proposed to detect the risk of flashover in contaminated insulators at an early stage [16]. The study contributed novelty to the literature by providing data collection methods. A CNN-based classification method based on infrared images has been proposed to determine the contamination level of insulator strings [17]. In the study, a general accuracy rate of 99.04% was achieved using the Softmax activation output function. Leakage currents obtained from dirty high-voltage insulators are classified into 5 different stages [18]. The study employs a deep learning architecture consisting of interconnected inception modules. The proposed model achieved a classification accuracy of over 95%.

Studies conducted using Fast Fourier Transform (FFT) and power spectrum density have shown that changes in icing amount and surface contamination affect the frequency distribution of the signal [19,20]. The data obtained through time–frequency analysis enhance the classification capability of advanced models such as convolutional neural networks by providing data-driven characterization of the insulator arc density [21]. These methods enable more precise monitoring of complex surface phenomena and the development of more reliable predictive models for icing-related problems [22].

This article proposes a spectrogram-based CNN model for the detection of glass, porcelain, and silicone insulators based on icing conditions. Glass (1, 2, and 3-unit), porcelain (1, 2, and 3-unit), and silicone insulators were tested under laboratory conditions for ice-free, slightly iced (t < 12 mm), and iced (t > 20 mm) conditions. According to the unit conditions, leakage current signals were obtained under three different voltage levels when high voltage was applied. Spectrogram images were obtained by applying the Fourier transform to the leakage current signals. After applying the Butterworth and smoothing filter to the spectrogram images, they were given as input to the developed CNN architecture. Spectrogram images were also applied to AlexNet, GoogLeNet, and ResNet-50 architectures. The developed CNN architecture demonstrated more stable classification performance across different insulator types and voltage levels compared with the AlexNet, GoogLeNet, and ResNet-50 architectures. The main contributions of this article can be summarized as follows.

(1) A comprehensive experimental dataset has been established to analyze the leakage current characteristics of porcelain, glass, and silicone insulators with single, double, and triple unit structures under varying icing conditions (ice-free, slightly iced, and iced) at voltage levels ranging from 10 to 50 kV.
(2) A two-stage preprocessing approach, comprising a Butterworth low-pass filter and a moving average smoothing filter, has been developed to eliminate noise in low-amplitude and rapidly varying leakage current data while keeping their distinguishing features.
(3) To process information in the time–frequency plane more efficiently, raw signals were converted into spectrogram images, and a novel CNN architecture incorporating Residual-Inception blocks was designed to enhance feature extraction capabilities.
(4) The proposed model demonstrated superior discriminative representation capability and generalization performance compared with established architectures in the literature, such as AlexNet, GoogLeNet, and ResNet-50, achieving accuracy rates of up to 100% across different voltage levels, particularly for glass and silicone insulators.

## 2. Analysis of Leakage Current Under Varied Thermal Conditions

The electrical performance of iced insulators is not affected only by parameters such as the thickness of the ice, the type of icing, and the length of the icicle [2]. The electrical conductivity of ice, which varies at different temperatures, also plays a decisive role in this performance. In the conducted studies, it was observed that ice conductivity increased significantly as the temperature rose from −15 °C to 0 °C. The majority of this change occurred within the narrow temperature range between −2 °C and 0 °C [23,24].

Leakage current is not only an electrical phenomenon but also an active factor that changes the thermal state of ice. Leakage current flowing through the ice layer or water film on the insulator causes Joule heating [23,25]. The heat generated by the leakage current forms a more conductive water film on the ice surface. The presence of this water film reduces the residual resistance on the insulator [26]. A decrease in resistance causes the leakage current to increase even more, and an increased current leads to more heating and melting. This condition causes electrical flashovers, negatively affecting the performance of the insulator.

### 2.1. Mathematical Model of the Effect of Ice on Insulator Leakage Current Behavior

The most common model used for ice-coated insulators is the Obenaus/Rizk approach [27,28]. According to this approach, the *V_m_* voltage is as expressed in Equation (1):

(1) $$V_{m}=AK_{p}xI_{m}^{-n}+I_{m}R(x)$$

where *Vm* is the peak value of the applied AC voltage (V), *Im* is the peak value of the AC current (A), *A* and *n* are the arc constants, *x* is the position of the arc root along the leakage path (cm), Kp is the ratio of the partial arc length in air to the arc root position, and *R(x)* is the resistance along the leakage path from position *x* to another (Ω).

The equation for the resistance *R(x)* in Equation (1) is expressed as in Equation (2).

(2) $$R\left(x\right)=\frac{10^{6}}{2\pi \gamma_{e}}\left(\frac{4(L-x)}{D+2t}+N\;In(\frac{D+2t}{4r})\right)$$

where $\gamma_{e}$ (T) is the equivalent surface conductivity (µS), *L* is the length of the arc path (cm), *x* is the position of the arc root along the leakage path (cm), *D* is the insulator diameter (cm), *t* is the ice thickness (cm), *N* is the total number of arc roots on the ice surface, and *r* is the arc root radius (cm).

The equivalent surface conductivity is expressed as in Equation (3).

(3) $$\gamma_{e}=\gamma_{bg}+{∆}_{\gamma }f_{m}(T)$$

where $\gamma_{bg}$ is the initial surface conductivity of the ice before melting, ${∆}_{\gamma }$ is the increase in conductivity added by melting and, $f_{m}(T)$ is the melt activation function.

The equation for the $\gamma_{bg}$ initial surface conductivity is as given in Equation (4) [29].

(4) $$\gamma_{bg}=h\sigma$$

where h is the thickness of the water film, and σ is the conductivity of the water film.

The Joule heat, calculated using the conductor’s resistance (*r_T_*), the current passing through it (*I*), and the conductor radius (*Rc*), is expressed as in Equation (5) [30].

(5) $$q=\frac{I^{2}r_{T}}{2\pi R_{c}^{2}}$$

In addition to the joule heat during melting in an icy insulator, it contributes to this process at different temperatures [25]. This situation is expressed as in Equation (6).

(6) $$Q_{n}+Q_{j}+Q_{s}-Q_{cq}-Q_{sr}-Q_{e}=0$$

where, $Q_{n}$ is the sum of the heat caused by sunlight and absorbed by the ice surface, $Q_{j}$ is the electrical heat caused by leakage current on the insulator surface, and $Q_{s}$ is the sum of other small heat sources in the process. $Q_{cq}$ refers to the heat transfer between the Ice surface and the air flow, $Q_{sr}$ refers to the loss of thermal radiation emitted from the ice surface to the outside, and $Q_{e}$ refers to the latent heat. Decayed during the evaporation of the ice.

### 2.2. Effect of Ice Melting on Leakage Current of Insulators Under High Voltage

The formation of ice on the insulator surface presents a substantial hazard for power systems, with the most crucial phase of this risk arising at the onset of the ice melting process. When the ice sheet begins to melt, a thin film of water forms on the surface [31]. Due to the crystallization effect, the water film contains dense ions. Therefore, the conductivity of meltwater increases rapidly and reaches a maximum level [32]. The higher the conductivity of the water, the lower the residual resistance of the ice on the surface, and the leakage current increases with a higher acceleration [33]. As the leakage current begins to flow through the water film, the heat generated by the electrical current (Joule heating) starts to melt the ice even further [34]. Leakage currents, which increase with the melting process, trigger the formation of an arc in the air gaps on the insulator surface. When these arcs exceed a critical threshold value, surface flashovers occur and can lead to energy outages.

**Figure 1:**
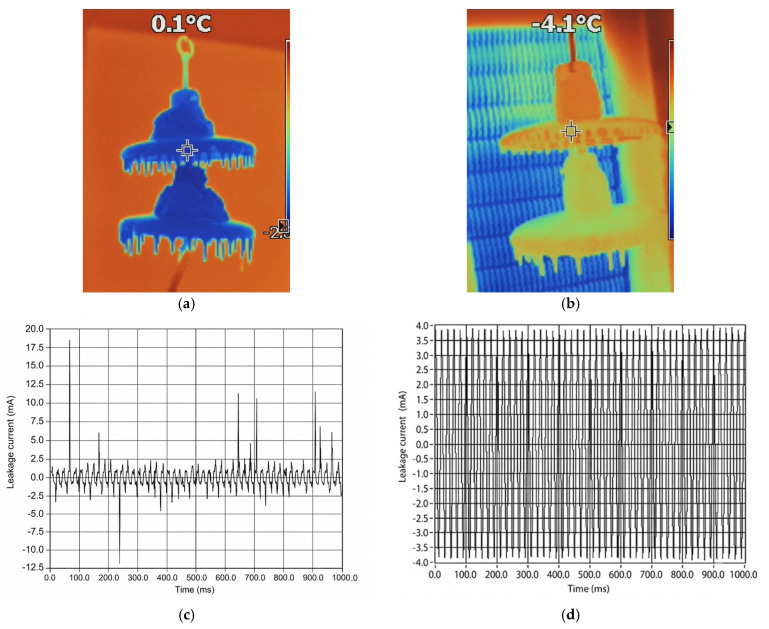
Temperature of the ice layer on the insulator surface (**a**,**b**) and the leakage currents obtained from the same conditions under applied high voltage (**c**,**d**).

Figure 1 shows the leakage current characteristics obtained under different ice temperatures with high voltage applied to the insulator surface. In Figure 1b, it is observed that the temperature of the ice on the insulator surface is −4.1 °C immediately after the insulator is removed from the icing chamber. The 1 s graph consisting of data taken at 1.5-millisecond intervals, corresponding to the 30 kV voltage applied at this value, is shown in Figure 1d. In Figure 1a, it is observed that after a period of time, the temperature of the ice on the insulator surface rises to 0.1 °C. Figure 1c shows a 1 s graph consisting of data taken at 1.5 ms intervals, corresponding to the 30 kV voltage applied at this value. The Fluke Ti100 model thermal camera((Fluke Corporation, Everett, WA, USA)) was used for non-contact measurement of temperature distributions and obtaining thermal images. The thermal camera has a refresh rate of 9 Hz, and the surface emissivity (ε) was adjusted to 0.92 for all measurements.

At −4.1 °C, the ice layer on the insulator surface forms a high surface resistance, resulting in the low-amplitude and stable sinusoidal leakage current waveform observed in Figure 1d. But when the temperature rises to 0.1 °C, surface melting leads to the formation of a conductive water film and an increase in surface conductivity ($\gamma_{e}$). This conductive layer triggers discharge processes as predicted by the Obenaus-Rizk model and carries the leakage current amplitude to dangerous peaks of approximately 18.5 mA.

The high-amplitude current pulses observed in Figure 1c indicate that the air gaps between the icicles are electrically stressed and that partial arcing has begun. In particular, irregular arcing events in the current graph reveal that the conductivity on the surface changes dynamically due to the effect of Joule heating, and this instability prepares the ground for insulation failure.

## 3. Materials and Methods

### 3.1. Experimental Setup

**Figure 2:**
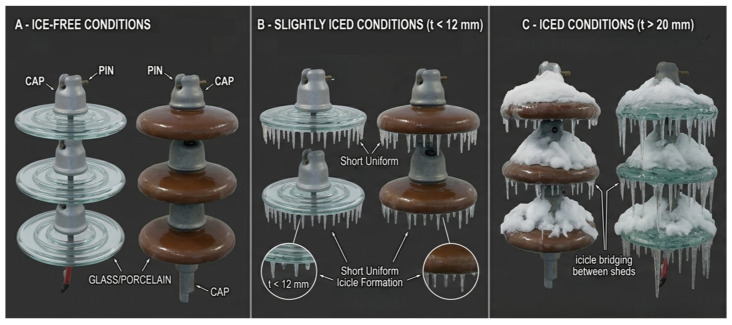
Comparative view of different icing intensities on insulators: (**A**) ice-free conditions, (**B**) slightly iced, (**C**) iced.

In this study, the icing process observed in insulators under operating conditions was experimentally reconstructed using accelerated methods in the laboratory environment. As can be seen in Equation (2), the thickness of the ice sheet is a determining factor in the formation of the leakage current [24]. For this reason, two main icing conditions were created in the study, slightly icing (t < 12 mm) and icing (t > 20 mm), taking into account the level of ice accumulated on the insulator surface. Figure 2 presents images of the insulators captured in the laboratory under the three icing conditions examined in this study.

As indicated in Equation (3), the surface conductivity of the insulator plays a critical role in the formation of leakage current. Therefore, prior to each experiment, the conductivity of the water sprayed onto the insulator surface before ice formation was measured using a conductivity meter to ensure controlled and consistent test conditions. The conductivity of the water used in the experiments was maintained within the range of 130–220 µS/cm. This range is consistent with the 100–200 µS/cm values recommended by the IEEE Insulator Icing Test Methods Task Group for the simulation of freezing rain scenarios in a laboratory setting [35] and also meets the requirements of IEEE Standard 1783-2009 [36]. While the recommended baseline water conductivity value for ice deposition tests is set at σ_20_ = 100 µS/cm, the same standard states that this value can be adjusted up or down based on field measurements [36]. On the other hand, it is stated that conductivity values exceeding σ_20_ = 300 µS/cm can lead to high leakage current density and discharge activity, causing premature flashover before ice deposition is complete [36]. In addition, it has been shown in the literature that extremely low conductivity values (≈2.5 µS/cm) suppress leakage current formation on the ice surface [37], while extremely high values increase the risk of early flashover and negatively affect experimental reliability. The selected conductivity range therefore represents a realistic and electrically meaningful test condition for evaluating the leakage current behavior due to icing.

**Figure 3:**
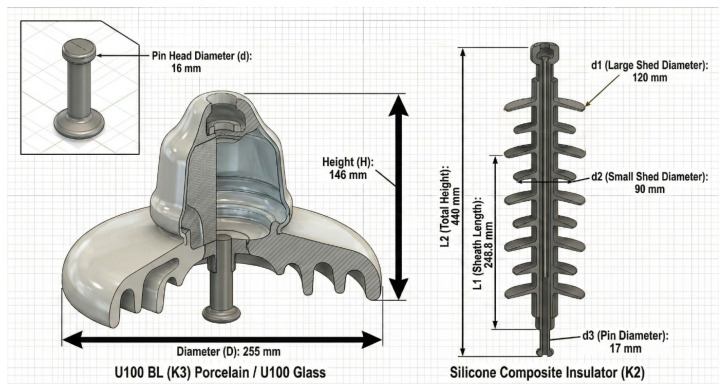
Cross-sectional views and characteristic values of porcelain, glass and silicone insulator models.

Figure 3 presents the cross-sectional views and characteristic parameters of the porcelain, glass, and silicone insulators used in this study.

The leakage current path length of porcelain and glass insulators is 280 mm, while the leakage current path length of the silicone insulator is 900 mm. For porcelain and glass insulators, the leakage current path length was increased by configuring the insulators as two- and three-unit assemblies. Depending on the resulting path length, voltage levels of 10, 20, and 30 kV were applied to single-unit porcelain and glass insulators; 20, 30, and 40 kV to two-unit configurations; and 30, 40, and 50 kV to three-unit configurations. For the silicone insulator, voltage levels of 30, 40, and 50 kV were applied. The variation in the applied voltage levels is attributed to differences in the leakage current path lengths of the insulator configurations. For each configuration, three distinct voltage levels were systematically selected to enhance dataset variability and to improve the robustness and generalization capability of the artificial intelligence-based model.

Considering the variations in insulator unit number, icing condition, and applied voltage levels, a total of 27 datasets were generated for porcelain insulators, 27 datasets for glass insulators, and 9 datasets for silicone insulators. Signal acquisition was performed using a National Instruments NI USB-6009 data acquisition (National Instruments, Austin, TX, USA)) (DAQ) module, featuring 14-bit resolution analog inputs and USB-based interfacing. The acquisition process was managed via LabVIEW ((version 2021) software to ensure synchronized, stable, and continuous data logging. For each dataset, 100,000 leakage current samples were collected. The sampling interval was approximately 1.5 ms, providing a temporal resolution suitable for capturing the icing-related leakage current behavior over long-duration recordings. The acquired data were directly transferred to a computer environment, archived in digital format, and prepared for advanced signal processing and deep learning analyses.

The performance of deep learning-based classification models strongly depends on the size and diversity of the training dataset. Larger datasets generally improve feature representation capability and enhance model generalization performance [38,39]. Therefore, acquiring 100,000 samples per dataset was considered sufficient to ensure robust model training while maintaining manageable computational complexity.

**Figure 4:**
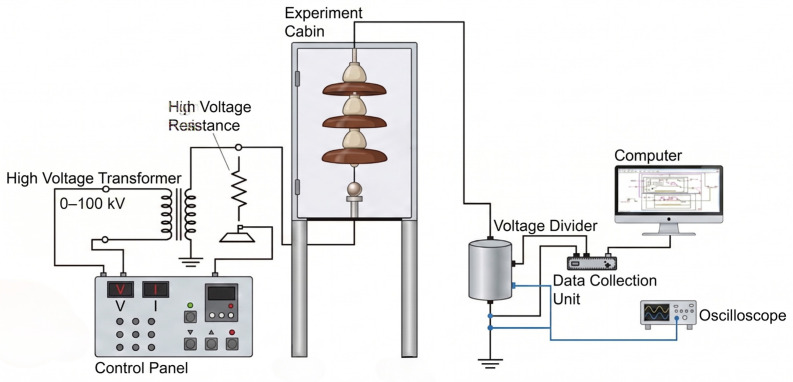
Experimental scheme used for measuring insulator leakage current under high voltage.

The experimental setup designed for leakage current analysis is shown in Figure 4.

The experimental studies were carried out on the test platform in Figure 4 in order to analyse the electrical changes in insulators with different icing levels. The main component of the system is a high-voltage transformer, controlled via a dedicated control panel, capable of providing an adjustable output in the range of 0–100 kV. To ensure operational safety and to limit leakage current, a high-voltage resistor was connected in series within the circuit. The data obtained from the iced insulator samples positioned vertically in the experiment cabinet are reduced to safe measurement levels via a voltage divider. The leakage current signals obtained from the insulator were simultaneously digitized using a data acquisition unit and subsequently transferred to a computer environment for further analysis. To process and analyze these signals, all computational and modeling operations were performed on a high-performance workstation. Experimental studies were conducted on a workstation equipped with an Intel Xeon w5-3433 processor, 128 GB of RAM, and an NVIDIA RTX A2000 graphics card with 12 GB of VRAM. MATLAB ((version R2025a) was employed as the software environment for signal processing and model implementation.

### 3.2. Signal Preprocessing

The signals recorded during the experimental studies were acquired by the data acquisition unit with a sampling period of 1.5 ms. The maximum representable frequency corresponding to this sampling interval was calculated based on the Nyquist sampling criterion, as expressed in Equation (7) [40].

(7) $$f_{Nyquıst}=\frac{1}{2T_{s}}=\frac{1}{2\times 1.5\times 10^{-3}\;}≅333\;Hz$$

The sampling frequency of the signal was calculated as in Equation (8).

(8) $$F_{s}=\frac{1}{T_{s}}=\frac{1}{1.5\times 10^{-3}}≅666.7\;Hz$$

This sampling rate was selected deliberately and was not constrained by the acquisition hardware, which supports considerably higher rates. The choice was guided by the physical nature of the phenomenon: the diagnostic information of the leakage current associated with surface conduction and discharge activity is concentrated in the fundamental component and the low-order odd harmonics, principally the third (150 Hz) and fifth (250 Hz) harmonics, both for contaminated insulators [41,42] and for ice-covered insulators during accretion and melting [43]. Both of these harmonics lie below the 333 Hz Nyquist frequency of the adopted sampling rate and are therefore fully resolved. In addition, since the icing severity develops through slow thermal and melting processes over tens of seconds, a long continuous record was prioritized to capture these transitions while keeping the data volume manageable.

Each raw signal recording comprises 100,000 samples, which at a sampling rate of 666.7 Hz corresponds to a duration of approximately 150 s. However, giving this long-term time series as input to direct machine learning or deep learning models increases the computational cost and may lead to instability in model training. Therefore, the signal was divided into fixed-length segments representing equal time intervals. In this study, each segment was designed to correspond to a 2 s time window, and the number of samples associated with this duration was determined based on the sampling frequency, as expressed in Equation (9).

(9) $$N_{window}=2\times 666\;≅1332$$

The windowed signal length was determined as 1332 samples, and no overlap was applied between consecutive segments. Accordingly, 75 windows were obtained from each recording of 100,000 samples. Since each insulator configuration comprises the recordings of its three icing conditions (ice-free, slightly iced, and iced), this results in 3 × 75 = 225 windows, and therefore 225 spectrogram images, for each configuration. Through this approach, each individual signal recording was transformed into multiple independent samples, thereby providing the substantial amount of data required for effective model training.

Each windowed segment was subjected to a two-stage noise reduction process prior to being fed into the model as input. In the first stage, a second-order Butterworth low-pass filter (BLPF) with a cutoff frequency of 300 Hz was applied to suppress high-frequency components originating from the sensor. The standard transfer function of a second-order Butterworth low-pass filter is shown as in Equation (10) [44].

(10) $$H\left(s\right)=\frac{{\omega_{c}}^{2}}{s^{2}+\sqrt{2}\;\omega_{c}s+{\omega_{c}}^{2}}$$

The amplitude transmission function of the BLPF is shown as in Equation (11) [44].

(11) $${\left|H(j\omega )\right|}^{2}=\frac{1}{1+{\left(\omega /{\;\omega }_{c}\right)}^{4}}$$

where, ${\left|H(j\omega )\right|}^{2}$ represents the squared magnitude of the filter’s frequency response, *j* = $\;\sqrt{-1}$, ω denotes the angular frequency, and $\omega_{c}$ corresponds to the cutoff frequency of the filter.

The upper cut-off frequency of the filter, 300 Hz, was selected taking into account the physical limits of the system. Since the Nyquist frequency is 333 Hz, components above 300 Hz cannot be represented correctly due to sampling requirements and are mainly characterized by sensor noise.

After filtering, a 6-sample moving average (MA) smoothing filter was applied as the second stage, since there may still be small amplitude fluctuations on the signal. The formula for the filter is shown in Equation (12) [45].

(12) $$y\left[n\right]=\frac{1}{6}\sum_{i=0}^{5}x[n-i]$$

where, *x* is the input signal and *y* is the output signal.

This window duration is approximately 9 ms (6/666) and only suppresses short-term local vibrations, without affecting the overall behaviour of the signal.

**Figure 5:**
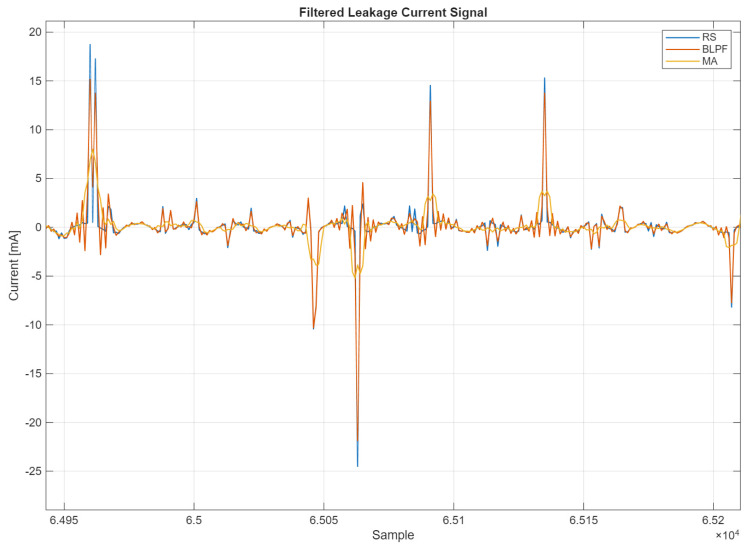
Comparison of raw leakage current signal (RS) with Butterworth low-pass filtered (BLPF) and moving average (MA) filtered waveforms.

Figure 5 shows filtering methods applied to a short time window of the dataset.

Figure 5 shows the blue raw signal, the red Butterworth filter, and the orange color smoothing filter.

Due to the high-frequency interference observed in the raw signal and noise components caused by measurement, a Butterworth low-pass filter with a cut-off frequency of 300 Hz was applied in the first stage. As a result of this filtering process, the high-frequency components are effectively suppressed, and a more stable structure is obtained, which more accurately reflects the basic characteristics of the signal. The moving average filter with a length of 6 samples preferred in the second stage reduced the short-term fluctuations and sudden amplitude changes remaining in the signal after the first filtering, resulting in a more uniform and stable output in the time domain.

### 3.3. Statistical Analysis

**Figure 6:**
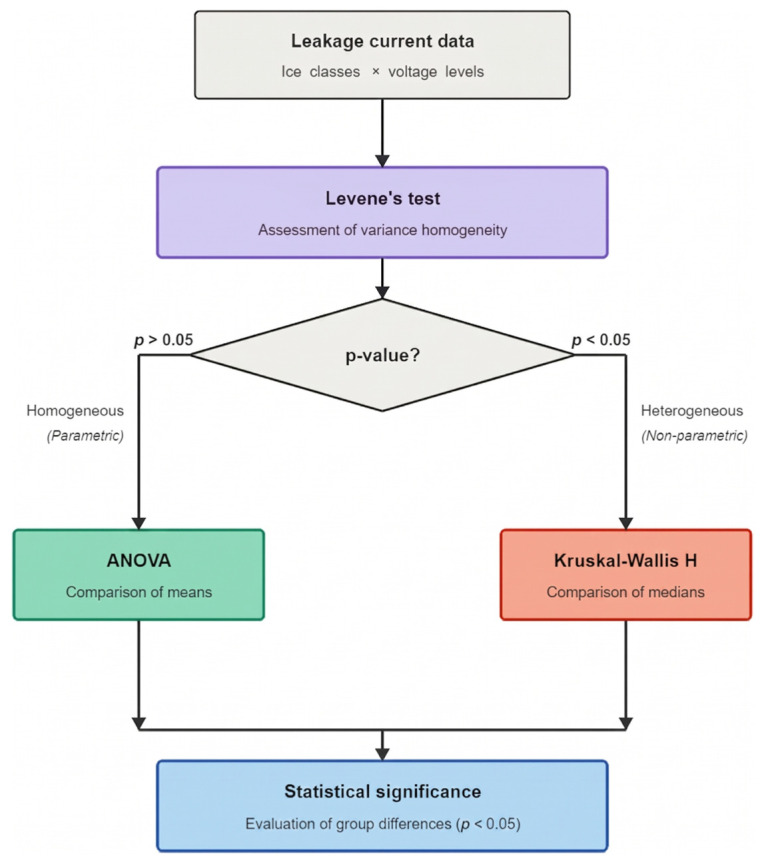
Flowchart of the statistical analysis procedure.

In scientific research, statistical methods are an important tool for identifying differences between datasets, establishing causal relationships, and validating hypotheses. The primary methods used, especially when comparing more than two groups, are Analysis of Variance (ANOVA) and its non-parametric alternative, the Kruskal–Wallis H test. The validity of these tests depends on whether the dataset satisfies certain statistical assumptions [46]. Accordingly, the Levene test is applied to assess the homogeneity of variances, and a parametric or non-parametric test is chosen based on the results obtained [47]. In statistical inference processes, the *p*-value (probability value) is an important criterion. If the Levene test result is *p* > 0.05, the variances are considered homogeneous, and the one-way ANOVA test is deemed applicable. Conversely, if *p* < 0.05, it is concluded that the variances are heterogeneous, and the non-parametric Kruskal–Wallis H test is preferred. The decision-making flowchart for selecting the appropriate statistical test is presented in Figure 6.

To determine the statistical significance between leakage current data, the homogeneity of variances obtained from ice-free, slightly icy, and icy conditions of the same material type was evaluated at each voltage level. If the variance is homogeneous, ANOVA test is used to analyze whether the leakage current values differ significantly in terms of their means. ANOVA tests whether the means statistically differ significantly between groups [48]. If the variances are not homogeneous, the Kruskal–Wallis test is used instead of ANOVA. The Kruskal–Wallis test is a non-parametric method that performs rank-based comparisons between groups using median values instead of means [49].

## 4. Spectrogram-Based Deep Convolutional Neural Network Approach for Leakage Current Detection in Ice-Covered Insulators

**Figure 7:**
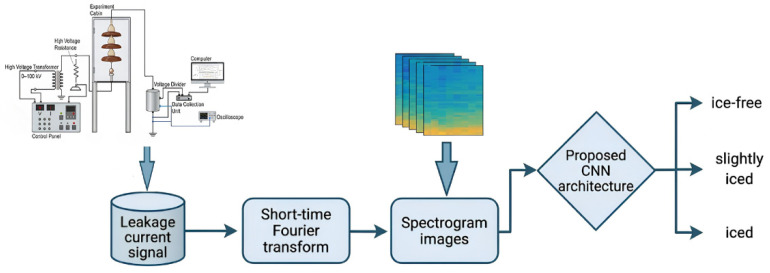
The deep learning-based framework proposed for monitoring and classifying insulator icing levels using leakage current signals.

The overall methodology, including the experimental measurement system, signal processing, and the classification stage using the proposed CNN architecture, is presented in Figure 7.

### 4.1. Spectrogram-Based Feature Extraction

Defects such as icing, contamination, and breakage/cracking on the insulator surface create harmonic distortions in leakage current signals, causing the frequency components of the signal to change over time. One of the methods commonly used to determine which frequency components a signal contains and when these components appear on the time axis is the Short-Term Fourier Transform (STFT) [50,51].

The leakage current signal is converted to the time–frequency (TF) domain, as in Equation (13) by applying the Short-Time Fourier Transform [52].

(13) $$X\left(\tau ,k\right)=\sum_{n=1}^{N}x\left[n\right]w\left[n-\tau \right]e^{-jnk}$$

where, x(n) is the signal to be transformed, and w(n) is the window function of length N. The spectrogram is the square of the Short-Time Fourier Transform and is given as in Equation (14):

(14) $$S\left(\tau ,k\right)=\;{\left|X\left(\tau ,k\right)\right|}^{2}$$

**Figure 8:**
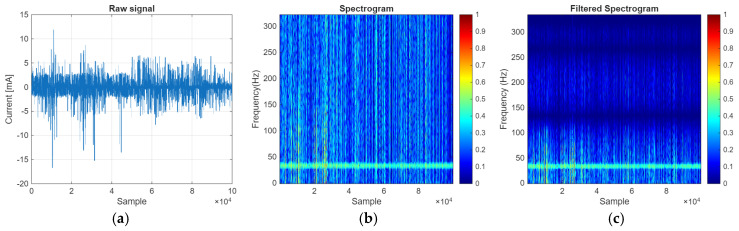
Raw signal (**a**), spectrogram (**b**) and (**c**) filtered spectrogram.

Spectrograms provide a visualization of the distribution of frequency components over time. This visualization helps to identify harmonics and other frequency-related distortions. Figure 8 shows the raw signal (Figure 8a), the corresponding spectrogram image (Figure 8b), and the filtered spectrogram image (Figure 8c).

In Figure 8a, distinct amplitude fluctuations and occasional high-amplitude sudden peaks are observed. This behavior can be attributed to irregular conductivity paths or surface discharges forming on the insulator surface. Figure 8b shows that the signal energy is largely concentrated in the low-frequency band, and higher energy densities occur at certain time intervals. These temporary increases in intensity indicate harmonic distortions or discharge events occurring in the leakage current signal. The spectrogram of the signal after two-stage filtering (BLPF and MA) is presented in Figure 8c. The implemented filtering process effectively suppressed background noise and high-frequency interference while preserving the essential components of the leakage current. The low-frequency band, clarified by suppressing noise components, allows for more precise diagnosis of the insulator condition by making the leakage current characteristics more distinct in the time–frequency domain.

### 4.2. Deep Convolutional Neural Network Architecture and Classification

Convolutional Neural Networks (CNNs) are multilayered, deep learning architectures widely used for extracting complex features [16,52]. These networks consist of hierarchical convolution and pooling layers designed to automatically filter and extract meaningful features from input data. In the analysis of complex signals in time series form, spectrograms that visualize the time-dependent spectral composition of the signal can be directly presented as input to CNN models. This approach, which converts the energy contents and variations of different frequencies in the signals into three-dimensional tensors, allows CNNs, which are superior in image processing, to learn the distinctive patterns in the signals much more easily [53]. These networks, which combine feature extraction and classification tasks in a single learning structure, generate the model’s final decision by transferring high-level features obtained from successive layers to fully connected layers in the final stage [54,55].

**Figure 9:**
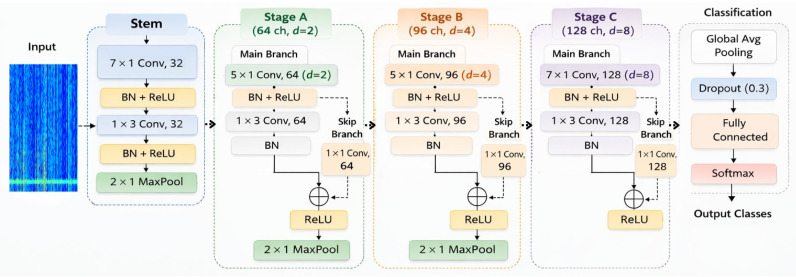
The proposed CNN architecture for detecting insulator icing conditions.

Figure 9 shows the CNN architecture containing the proposed Residual-Inception blocks for insulator icing condition detection.

The CNN architecture shown in Figure 9 consists of a stem block, three consecutive residual learning stages (Stage A, Stage B, and Stage C), and a classification block. In the stem block, asymmetric convolutions with kernel sizes of 7 × 1 and 1 × 3 were employed to separately capture discriminative vertical and horizontal patterns from the input, followed by a 2 × 1 max-pooling layer for spatial downsampling. This structure enables more effective learning of first-level features, particularly in input representations that contain narrow-band and directional information.

In the three residual stages following the stem block, the number of channels was gradually increased to 64, 96, and 128; conversely, the dilation coefficients were increased to 2, 4, and 8, respectively. At each stage, dilated convolution-based asymmetric filtering was applied to the main branch, while dimensional mapping was performed on the lateral branch using 1 × 1 convolution, and the two branches were joined via the residual connection. This residual structure improves gradient flow, providing more stable training; increased dilation coefficients expand the receiver area, allowing for the learning of larger-scale contextual information. Thus, architecture is able to represent both local fine details and broader spatial dependencies within the same network structure.

In the final stage, the resulting high-level feature maps were fed into the global average pooling layer, then regularization was applied with a 30% dropout, and class probabilities were generated via the fully coupled layer. The final three-class decision was made using a Softmax-based output structure. This architecture offers a balanced framework in terms of parameter efficiency, training stability, and multi-scale feature representation.

Table 1 provides a description of the network architecture used.

The table shows the core sizes, stride values, dilation coefficients, and the dimensions of the resulting feature maps used at each stage of the network. Choosing an asymmetric kernel in the initial stages of the architecture minimizes computational cost while enabling the separation of directional features in the input matrix. Increasing channel capacity from Stage A to Stage C strengthens the network’s abstraction capability, while simultaneously increasing the dilation factors logarithmically expands the model. This structure allows the correlation between local pixels and distant data points to be modeled more efficiently as the network depth increases. Furthermore, the dimensional alignment between the main branches and the skip branches is managed through a 1 × 1 convolutional mapping process, preventing losses in information transmission. This CNN architecture allows the model to learn features at different scales while maintaining the computational efficiency of the network.

### 4.3. Performance Evaluation Metrics

The performance evaluation of the proposed model for classifying insulator icing levels is carried out by comparing the model’s predictions with the actual labels. This situation is defined through four basic components [56,57]. True Positives (*TP*) refer to cases where the model correctly identifies the icing condition on the insulator. True Negatives (*TN*) are the correct identification of an ice-free insulator. False Positives (*FP*) are cases where an ice-free insulator is incorrectly reported. False Negatives (*FN*) occur when the iced insulator is ignored by the system. Commonly used metrics such as accuracy, sensitivity, specificity, precision, and F1-score were used to evaluate the classification performance.

Accuracy measures how many of the positive and negative observations the model correctly classifies within all the data it examines. In other words, it expresses the percentage of correctly predicted observations relative to the total number of cases, as given in Equation (15).

(15) $$Accuracy=\frac{TP+TN}{TP+TN+FP+FN}$$

Sensitivity indicates how many of all observations that are actually positive were successfully predicted as positive by the model. This metric is used to evaluate the model’s ability to find all positive cases in the dataset and is expressed as in Equation (16).

(16) $$Sensitivity=\frac{TP}{TP+FN}$$

Specificity measures how many of all observations that are actually negative are correctly classified as negative by the model. This metric shows the model’s ability to distinguish negative cases and is expressed as in Equation (17).

(17) $$Specificity=\frac{TN}{TN+FP}$$

Precision refers to how many of the observations predicted positively by the model are actually correct. This metric measures the extent to which the model makes the error of mistakenly perceiving a negative signal as positive and is expressed as in Equation (18).

(18) $$Precision=\frac{TP}{TP+FP}$$

The F1-score is a performance measure representing the harmonic mean of the Precision and Sensitivity values and is expressed as in Equation (19).

(19) $$F1-score=\frac{2\times Sensitivity\times Precision}{Sensitivity+Precision}$$

## 5. Discussion

### 5.1. Data Validation and Statistical Characterization

Glass, porcelain, and silicone-based insulator types were tested under varying icing intensities and voltage levels depending on the number of units. The statistical significance of the 63 datasets obtained at the end of this experimental process is of critical importance in terms of the reliability and comparability of the findings. In the analysis of the datasets, icing conditions at each voltage level were organized into 1 × 3 matrix structures. Based on the number of insulator units (1U, 2U, and 3U), this classification resulted in 9 matrix data points each for porcelain and glass insulator groups, and 3 matrix data points for the silicone insulator group. The results of the Levene homogeneity of variance, ANOVA, and Kruskal–Wallis tests, which were applied to determine the statistical distribution characteristics and intergroup differences of the generated datasets, are presented in Table 2.

Levene test results show that the three insulator types examined did not satisfy the assumption of variance homogeneity at most voltage levels. This situation reveals that leakage current distributions are heterogeneous and potentially non-linear in character. Therefore, parametric ANOVA results were evaluated together with the non-parametric Kruskal–Wallis test.

In almost all groups of porcelain, glass, and silicone insulators, the *p*-values obtained from both ANOVA and Kruskal–Wallis tests are much smaller than 0.05. In particular, the *p* < 0.001 values obtained for all voltage levels in silicone insulators indicate that the response of this material type to icing is more pronounced and distinctive compared with other groups. The high *p*-values observed in some specific cases, such as 30 kV–2U, in glass insulators indicate that the difference in icing levels is statistically less significant in this configuration.

The findings reveal that leakage current characteristics can vary significantly under different insulator types and operating conditions. This indicates that using both parametric and non-parametric statistical methods together in the analysis processes is an appropriate approach. Furthermore, the results show that leakage current behavior is significantly affected by both the applied voltage level and the insulator configuration.

### 5.2. Performance Evaluation and Comparative Analysis of CNN Architectures

Experimental studies were conducted by creating three different icing conditions on porcelain, glass, and silicone insulators: ice-free, slightly iced, and iced. Three different voltage levels ranging from 10 to 50 kV were applied depending on the insulator configuration, and a dataset was obtained from the leakage current values generated under these conditions. To demonstrate the reliability and generalizability of the proposed CNN model for dataset classification, a comprehensive evaluation of the model’s performance is necessary. The performance of the developed CNN-based model was evaluated using key performance metrics such as accuracy, precision, sensitivity, specificity, and F1-score. The performance metrics obtained are shown in Table 3.

The recommended model in the glass and silicone insulator groups has demonstrated stable and high performance, ranging from 0.9778 to 1.0000, proving its ability to distinguish icing conditions with high accuracy. In most configurations for porcelain insulators, accuracy values above 0.90 have been achieved. However, the accuracy value in classifying leakage currents obtained from a 50 kV voltage applied to a three-unit porcelain insulator decreased to 0.8222. This situation suggests that the resulting leakage current spectrograms may exhibit a more complex structure. Specifically, under this most severe configuration, the combination of heavy icing and a high applied field reduces the spectral distinction between adjacent icing classes, so that the spectrograms of the slightly iced and iced states become more similar and partially overlap, which lowers their inter-class separability. The test results showing a generally high level of consistency between F1-score values and accuracy metrics confirm that the proposed deep learning approach has a high generalization ability in distinguishing classes in the dataset and that the model exhibits a balanced performance between precision and sensitivity.

**Figure 10:**
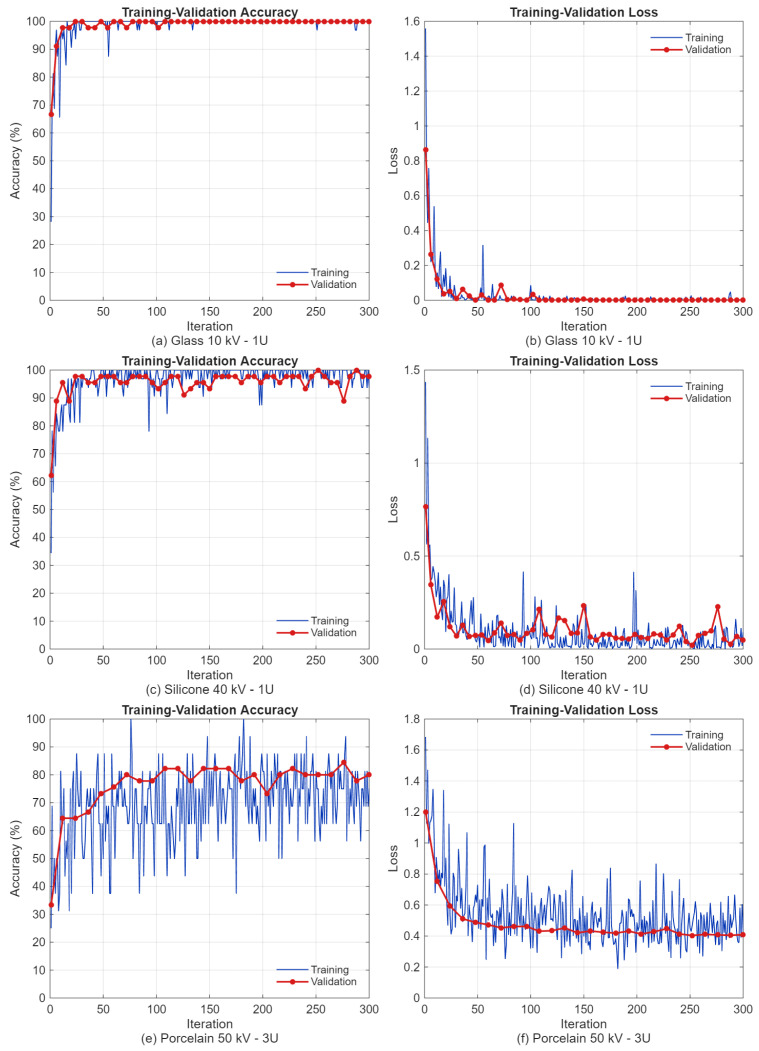
Training and validation accuracy and loss curves for Glass 10 kV–1U (**a**,**b**), Silicone 40 kV–1U (**c**,**d**), and Porcelain 50 kV–3U (**e**,**f**).

Figure 10 presents the training and validation accuracy and loss curves for Glass 10 kV–1U (Figure 10a,b), Silicone 40 kV–1U (Figure 10c,d), and Porcelain 50 kV–3U (Figure 10e,f).

The dataset for each insulator configuration consists of 225 spectrogram images. In total, 80% of these data were used to train the model, and the remaining 20% was used to test the performance of the trained model. This split was performed at the image level separately for each class, and since the windows are non-overlapping, no raw signal samples are shared between any of them. A light on-the-fly data augmentation, consisting of small random translations of at most two pixels vertically and one pixel horizontally with no rotation or reflection, was applied during training. This augmentation was applied only to the training partition after the split, while the held-out test set was never augmented; consequently, no data leakage occurs between the training and test sets. The proposed CNN model was trained with the Adam optimization algorithm, an initial learning rate of 0.0003, and 60 epochs. As shown in Figure 10a, the model exhibits a very rapid convergence, reaching 100% accuracy in approximately 20–25 iterations. Although slight fluctuations are observed in Figure 10c, the training and validation curves followed a consistent course, and a high validation accuracy of 97.78% was achieved. In the more complex spectrogram group presented in Figure 10e, more pronounced oscillations were observed during the training and validation processes. Nevertheless, the model achieved an accuracy rate of 82.22%, and the resulting training curves show that the proposed CNN architecture exhibits a learning performance of over 80% even under challenging conditions.

Table 4 compares the proposed CNN model with other models.

The proposed CNN architecture in Table 4 achieved 100% classification accuracy for the glass insulators, performing at least as well as the baseline models, which ranged from 84.44% to 100%. In cases involving porcelain insulators and more complex spectrogram images such as 50 kV–3U, the accuracy rates of the ResNet-50 and GoogLeNet models were 73.33% and 77.78%, respectively. The proposed model demonstrated superior performance with an accuracy rate of 82.22%. In the case of silicone insulators, the accuracy rates of the AlexNet, GoogLeNet, and ResNet-50 models were 2–11% lower compared with the recommended model.

## 6. Conclusions

This study presents a dataset generated from leakage current data obtained from iced insulator surfaces and a deep learning architecture for classifying insulator icing conditions. A CNN architecture designed to classify three different icing conditions of insulators (ice-free, slightly iced, and iced) was used. By increasing the number of units in glass, porcelain, and silicone insulators, configurations with different leakage current paths were created, and leakage current data were obtained at three different voltage levels. This approach contributed to more effective training of the artificial intelligence model by increasing the diversity and number of datasets. Thus, the recognition of leakage current characteristics in insulators made of different materials and at different voltage levels by artificial intelligence has the potential to offer a faster and more effective solution for detecting icing conditions in future studies.

The statistical results show that leakage current characteristics vary significantly not only depending on the applied voltage level, but also on the insulator type and number of units. This highlights the need for a multivariate analysis approach in evaluating the performance of different insulator configurations under high-voltage conditions. Furthermore, the combined use of parametric and non-parametric tests allowed for a more comprehensive evaluation of the statistical properties of the dataset and increased the reliability of the results obtained. This approach contributes to a more robust and consistent assessment in the comparative analysis of leakage current data obtained under different operating conditions.

To reduce noise effects that may occur during the acquisition of leakage current data, filtering was applied to the signals. Subsequently, these signals were converted into spectrogram images using the Short-Time Fourier Transform (STFT) to create a more meaningful data representation in the learning process of the proposed CNN architecture and presented as input to the model. The results show that the proposed architecture provides improvements in accuracy performance of up to 13.34% in porcelain, 15.56% in glass, and 13.33% in silicone insulators when compared with commonly used deep learning architectures such as AlexNet, GoogLeNet, and ResNet-50. These findings demonstrate that analyzing leakage current-based spectrogram data with deep learning methods offers an effective approach for reliably detecting insulator icing conditions.

This study is pioneering in developing signal processing-based approaches for the early detection of icing problems in insulators and in expanding research in this field. Future research should aim to move beyond laboratory settings for the early detection of icing conditions on insulators and develop early warning systems with systematically defined parameters.

## Figures and Tables

**Table 1:** Structure of the proposed architecture for classifying iced insulator leakage current signals.

Stage	Layer Configuration	Kernel/Stride/Dilation	Output Size
Input	Spectrogram image	–	256 × 8 × 3
Stem	Conv + BN + ReLU	7 × 1/2 × 1/1	128 × 8 × 32
	Conv + BN + ReLU	1 × 3/1 × 1/1	128 × 8 × 32
	MaxPooling	2 × 1/2 × 1	64 × 8 × 32
Stage A	Conv + BN + ReLU	5 × 1/1 × 1/2	64 × 8 × 64
	Conv + BN	1 × 3/1 × 1/1	64 × 8 × 64
	Skip connection (1 × 1 Conv)	1 × 1/1 × 1	64 × 8 × 64
	Residual addition + ReLU	–	64 × 8 × 64
	MaxPooling	2 × 1/2 × 1	32 × 8 × 64
Stage B	Conv + BN + ReLU	5 × 1/1 × 1/4	32 × 8 × 96
	Conv + BN	1 × 3/1 × 1/1	32 × 8 × 96
	Skip connection (1 × 1 Conv)	1 × 1/1 × 1	32 × 8 × 96
	Residual addition + ReLU	–	32 × 8 × 96
	MaxPooling	2 × 1/2 × 1	16 × 8 × 96
Stage C	Conv + BN + ReLU	7 × 1/1 × 1/8	16 × 8 × 128
	Conv + BN	1 × 3/1 × 1/1	16 × 8 × 128
	Skip connection (1 × 1 Conv)	1 × 1/1 × 1	16 × 8 × 128
	Residual addition + ReLU	–	16 × 8 × 128
Classification head	Global Average Pooling	–	1 × 1 × 128
	Dropout	*p* = 0.3	1 × 1 × 128
	Fully Connected	3 neurons	1 × 1 × 3
Output	Softmax classifier	3 classes	1 × 1 × 3

**Table 2:** Statistical *p*-values for leakage current data under different voltage levels and insulator configurations.

Insulator Type	Voltage (kV)–Number of Units	Levene *p*-Value	ANOVA *p*-Value	Kruskal–Wallis *p*-Value
Porcelain	10 kV–1U	2.018 × 10^−213^	0.0443	5.1435 × 10^−5^
Porcelain	20 kV–1U	3.640 × 10^−148^	3.7996 × 10^−6^	0.0554
Porcelain	30 kV–1U	<0.001	1.3194 × 10^−5^	0.0012
Porcelain	20 kV–2U	0.0554	3.7996 × 10^−6^	0.0554
Porcelain	30 kV–2U	6.0411 × 10^−8^	0.0133	6.0411 × 10^−8^
Porcelain	40 kV–2U	6.5799 × 10^−18^	0.0025	6.5799 × 10^−18^
Porcelain	30 kV–3U	1.4735 × 10^−7^	0.3392	1.4735 × 10^−7^
Porcelain	40 kV–3U	1.8699 × 10^−32^	0.0163	1.8699 × 10^−32^
Porcelain	50 kV–3U	4.5896 × 10^−6^	2.7188 × 10^−9^	4.5896 × 10^−6^
Glass	10 kV–1U	<0.001	2.0547 × 10^−7^	0.3269
Glass	20 kV–1U	<0.001	<0.001	2.8110 × 10^−131^
Glass	30 kV–1U	<0.001	5.1427 × 10^−12^	6.0602 × 10^−31^
Glass	20 kV–2U	2.8110 × 10^−131^	<0.001	2.8110 × 10^−131^
Glass	30 kV–2U	0.1597	0.8588	0.1597
Glass	40 kV–2U	1.2168 × 10^−12^	1.7831 × 10^−5^	1.2168 × 10^−12^
Glass	30 kV–3U	0.0092	0.2339	0.0092
Glass	40 kV–3U	3.1220 × 10^−10^	5.5993 × 10^−25^	3.1220 × 10^−10^
Glass	50 kV–3U	1.8433 × 10^−49^	3.0464 × 10^−93^	1.8433 × 10^−49^
Silicone	30 kV–1U	<0.001	1.5212 × 10^−8^	6.2293 × 10^−34^
Silicone	40 kV–1U	<0.001	5.8716 × 10^−12^	4.0739 × 10^−79^
Silicone	50 kV–1U	<0.001	2.2652 × 10^−103^	9.2207 × 10^−204^

**Table 3:** Performance evaluation metrics of the proposed CNN model.

Insulator Type	Voltage (kV)–Number of Units	Accuracy	Sensitivity	Specificity	Precision	F1-Score
Porcelain	10 kV–1U	0.9778	0.9778	0.9889	0.9792	0.9778
Porcelain	20 kV–1U	0.9778	0.9778	0.9889	0.9792	0.9778
Porcelain	30 kV–1U	0.9333	0.9333	0.9667	0.9345	0.9333
Porcelain	20 kV–2U	0.9111	0.9111	0.9556	0.9298	0.9095
Porcelain	30 kV–2U	1.0000	1.0000	1.0000	1.0000	1.0000
Porcelain	40 kV–2U	0.8889	0.8889	0.9444	0.9167	0.8857
Porcelain	30 kV–3U	0.8889	0.8889	0.9444	0.9167	0.8857
Porcelain	40 kV–3U	0.9333	0.9333	0.9667	0.9345	0.9333
Porcelain	50 kV–3U	0.8222	0.8222	0.9111	0.8250	0.8214
Glass	10 kV–1U	1.0000	1.0000	1.0000	1.0000	1.0000
Glass	20 kV–1U	1.0000	1.0000	1.0000	1.0000	1.0000
Glass	30 kV–1U	1.0000	1.0000	1.0000	1.0000	1.0000
Glass	20 kV–2U	1.0000	1.0000	1.0000	1.0000	1.0000
Glass	30 kV–2U	1.0000	1.0000	1.0000	1.0000	1.0000
Glass	40 kV–2U	1.0000	1.0000	1.0000	1.0000	1.0000
Glass	30 kV–3U	1.0000	1.0000	1.0000	1.0000	1.0000
Glass	40 kV–3U	0.9333	0.9333	0.9667	0.9345	0.9333
Glass	50 kV–3U	0.9778	0.9778	0.9889	0.9792	0.9778
Silicone	30 kV–1U	0.9778	0.9778	0.9889	0.9792	0.9778
Silicone	40 kV–1U	0.9778	0.9778	0.9889	0.9792	0.9778
Silicone	50 kV–1U	1.0000	1.0000	1.0000	1.0000	1.0000

**Table 4:** Comparison of the proposed CNN model with established models in the literature.

Insulator Type	Voltage (kV)–Number of Units	Proposed CNN Model	AlexNet	GoogLeNet	ResNet-50
Porcelain	10 kV–1U	0.9778	0.8444	0.8889	0.8889
Porcelain	20 kV–1U	0.9778	0.9111	0.9111	0.9333
Porcelain	30 kV–1U	0.9333	0.8667	0.9333	0.9111
Porcelain	20 kV–2U	0.9111	0.8667	0.8222	0.8889
Porcelain	30 kV–2U	1.0000	0.9777	0.9111	0.9778
Porcelain	40 kV–2U	0.8889	0.8667	0.8444	0.7556
Porcelain	30 kV–3U	0.8889	0.8000	0.8000	0.8000
Porcelain	40 kV–3U	0.9333	0.8444	0.9333	0.9333
Porcelain	50 kV–3U	0.8222	0.8000	0.7778	0.7333
Glass	10 kV–1U	1.0000	1.0000	1.0000	1.0000
Glass	20 kV–1U	1.0000	1.0000	1.0000	1.0000
Glass	30 kV–1U	1.0000	0.8889	0.8444	0.9778
Glass	20 kV–2U	1.0000	1.0000	0.9778	0.9778
Glass	30 kV–2U	1.0000	1.0000	0.9556	1.0000
Glass	40 kV–2U	1.0000	1.0000	0.9556	0.9333
Glass	30 kV–3U	1.0000	0.9556	0.8889	0.9111
Glass	40 kV–3U	0.9333	0.9111	0.8667	0.9556
Glass	50 kV–3U	0.9778	0.9111	0.9111	0.9333
Silicone	30 kV–1U	0.9778	0.8889	0.9556	0.8889
Silicone	40 kV–1U	0.9778	0.9556	0.9778	0.9556
Silicone	50 kV–1U	1.0000	0.8889	0.8667	0.9111

## Data Availability

The data presented in this study are available on request from the corresponding author. The data are not publicly available at this time as they form part of an ongoing study.
